# Perinatal Outcome and Long-Term Gastrointestinal Morbidity of Offspring of Women with Celiac Disease

**DOI:** 10.3390/jcm8111924

**Published:** 2019-11-08

**Authors:** Avishag Abecassis, Tamar Wainstock, Eyal Sheiner, Gali Pariente

**Affiliations:** 1 Department of Obstetrics and Gynecology, Soroka University Medical Center, Ben-Gurion University of the Negev, Beer-Sheva 8410501, Israel; avishaga3@gmail.com (A.A.); galipa@bgu.ac.il (G.P.); 2 Faculty of Health Sciences, Ben-Gurion University of the Negev, School of Public Health, Beer-Sheva 8410501, Israel; wainstoc@post.bgu.ac.il

**Keywords:** celiac, gastrointestinal morbidity, long term, maternal celiac, offspring

## Abstract

The aim of this study was to evaluate perinatal outcome and long-term offspring gastrointestinal morbidity of women with celiac disease. Perinatal outcomes, as well as long-term gastrointestinal morbidity of offspring of mothers with and without celiac disease were assessed. The study groups were followed until 18 years of age for gastrointestinal-related morbidity. For perinatal outcomes, generalized estimation equation (GEE) models were used. A Kaplan–Meier survival curve was used to compare cumulative incidence of long-term gastrointestinal morbidity, and Cox proportional hazards models were constructed to control for confounders. During the study period, 243,682 deliveries met the inclusion criteria, of which 212 (0.08%) were to mothers with celiac disease. Using GEE models, maternal celiac disease was noted as an independent risk factor for low birth weight and cesarean delivery. Offspring born to mothers with celiac disease had higher rates of gastrointestinal related morbidity (Kaplan–Meier log rank test *p* < 0.001). Using a Cox proportional hazards model, being born to a mother with celiac disease was found to be an independent risk factor for long-term gastrointestinal morbidity of the offspring. Pregnancy of women with celiac disease is independently associated with adverse perinatal outcome as well as higher risk for long-term gastrointestinal morbidity of offspring.

## 1. Introduction

Celiac disease, a gluten sensitive enteropathy, is a chronic autoimmune disorder affecting the normal villous structure in the small intestine. The clinical picture of celiac disease is being triggered by environmental exposure to gliadin in genetically predisposed individuals with human leukocyte antigen (HLA) DQ2/8 [1,2]. The classic disease presents features such as villous atrophy, mucosal inflammation, diarrhea, weight loss, and nutrient or vitamin deficiency due to malabsorption, while patients with atypical disease exhibit nonspecific gastrointestinal symptoms without signs of malabsorption, or with extra-intestinal manifestations such as anemia, osteoporosis, dental enamel hypoplasia, arthritis, and neurological symptoms [2,3,4]. The development and availability of diagnosis has made the prevalence of celiac disease increase over the past 20 years [5]. The prevalence of celiac disease in western countries is around 1% of the general population; however, significant numbers of patients with celiac disease are still undiagnosed [3].

Several studies have investigated short-term pregnancy outcomes of women with celiac disease [5,6]. Recurrent pregnancy loss, preterm delivery [7,8], intrauterine growth restriction (IUGR) [9,10], and low birth weight [11] are some of the obstetric complications that have been demonstrated to be associated with celiac disease. These pregnancy complications associated with celiac raise the question of importance of celiac screening during pregnancy [5].

Less is known regarding long-term gastrointestinal outcome and prevalence of celiac disease among offspring of mothers with celiac disease [12]. Genetic predisposition plays an important role in the prevalence of celiac disease, in first- and second-degree relatives, regardless of gastrointestinal symptoms [13]. Furthermore, a meta-analysis that included 54 articles showed that 7.5% of first-degree relatives and 2.3% of second-degree relatives of patients with celiac disease have celiac disease themselves. The highest prevalence demonstrated was in siblings (8.9%), followed by offspring (7.9%) [14].

Therefore, we sought to investigate the short-term perinatal outcome of mothers with celiac disease as well as to further evaluate the association between gastrointestinal morbidity and specifically celiac disease in offspring of mothers with celiac disease.

## 2. Materials and Methods

### 2.1. Study Design

A population-based retrospective cohort study was conducted. The study investigated perinatal outcomes of mothers with celiac disease and long- term morbidity of offspring of mothers with celiac disease. Offspring of mothers without celiac disease comprised the comparison (unexposed) group.

Pregnancy characteristics, perinatal outcomes, and long-term gastrointestinal morbidity of the offspring were compared between offspring of mothers with and without celiac disease. Perinatal outcomes such as maternal diabetes mellitus, hypertensive disorders, placental abruption, mode of delivery, low birth weight, low Apgar scores, and perinatal mortality were assessed.

Long-term gastrointestinal morbidity was evaluated. Gastrointestinal morbidity assessed included hospitalizations of the offspring up to the age of 18 years with any of the following diagnoses: esophageal disease, gastro-duodenal disease, appendix disease, ano-rectal disease, umbilical or inguinal hernia, inflammatory bowel diseases (IBD), functional colon disease, hemorrhoids, and celiac disease. The predefined ICD-9 code list of all diagnoses included in each of these conditions is detailed in the supplemental table (Supplemental Table S1). Follow-up time was defined as time to an event (hospitalization with any gastrointestinal diagnosis). Follow-up was terminated if any of the following occurred: first hospitalization with any gastrointestinal diagnosis (i.e., an event), hospitalization resulting in death, or when the child reached 18 years of age.

### 2.2. Settings and Study Population

The study was conducted at the Soroka University Medical Center, a tertiary medical center and the only hospital in southern Israel that occupies 65% of Israel’s territory (approximately 1.22 million people) [15]. The institutional review board, in accordance with the Helsinki declaration, approved the study (# SOR-0220-17).

All singleton pregnancies of women who delivered between the years 1991 and 2014 at the Soroka University Medical Center were included in the study. Multiple pregnancy and children with congenital malformations or chromosomal abnormalities were excluded from the study. Cases of perinatal deaths were excluded from the long-term follow-up cohort.

### 2.3. Data Collection Methods

Data were collected from two databases that were crosslinked and merged, based on mothers’ and infants’ identification numbers: the computerized perinatal database of the Obstetrics and Gynecology department and general Soroka University Medical Center computerized hospitalization database (“Demog-ICD9”). The obstetrical database includes demographic information, perinatal assessments, maternal morbidities, and maternal and fetal outcomes and is recorded immediately following delivery by the attending physician. Records were anonymized prior to analysis. The general Soroka University Medical Center hospitalization database includes demographic information and international classification of diseases, ninth revision codes (ICD-9), for all medical diagnosis made during any hospitalization. All newborns are issued with a national security number (ID number), which is then registered in the mother’s formal identification card. These identification numbers are not changed nor duplicated within the population at any given time. This allowed us to be certain of the relationship between any mother and child in our datasets.

### 2.4. Statistical Analysis

Statistical analysis was performed using SPSS (version 23). Univariate analysis was performed to compare dependent and background characteristics between the two study groups. Differences in categorical data were assessed by chi-square for general association. The *t-*test was used for comparison of continuous variables with normal distribution. To compare perinatal outcomes, generalized estimation equation (GEE) models were used to control for confounders and to account for maternal clusters. A Kaplan–Meier survival curve was used to compare cumulative gastrointestinal related hospitalization incidences over time according to celiac exposure. The difference between the two cumulative morbidity curves was assessed using the long rank test. A Cox proportional hazard model analysis was used to establish an independent association between maternal celiac during pregnancy and future gastrointestinal related hospitalization risk while controlling for potential confounders. All analyses were two sided, and a *p*-value of less than 0.05 was considered statistically significant.

## 3. Results

### 3.1. Maternal Characteristics

The study population included 243,682 deliveries which met the inclusion criteria, among them 212 (0.08%) neonates born to mothers with known celiac disease.

Maternal demographic characteristics are summarized in Table 1. No significant differences were noted in maternal age, parity, and ethnicity between mothers with and without celiac disease.

### 3.2. Maternal Celiac Disease and Adverse Perinatal Outcome

Higher rates of perinatal complications were noted among women with celiac disease; preterm delivery (10.4% vs. 6.9, *p* = 0.043), cesarean delivery (18.4% vs. 13.5%, *p* = 0.039), and low birth weight (14.2% vs. 6.7%, *p* < 0.001) were all higher among women with celiac disease compared to the control group. Using GEE models, controlling for maternal age and parity, maternal celiac disease was noted as an independent risk factor for low birth weight (adjusted OR 2.2, 95% CI 1.50–3.47, *p* ≤ 0.001) and cesarean delivery (adjusted OR 1.4, 95% CI 1.02–2.07, *p* = 0.035, Table 2).

### 3.3. Maternal Celiac Disease and Long-Term Gastrointestinal Morbidity of the Offspring

**Figure 1:**
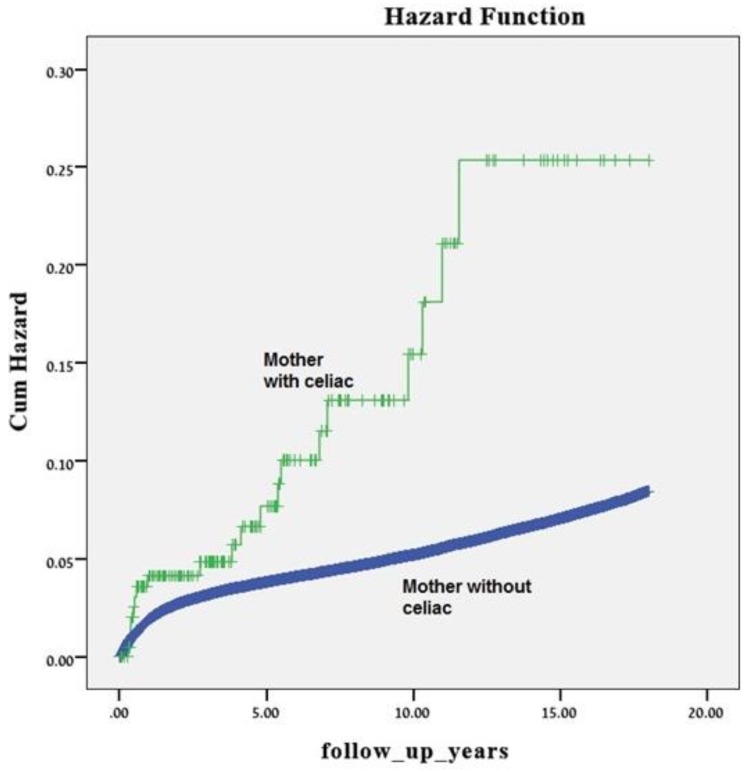
A Kaplan–Meier survival curve demonstrating the cumulative incidence of long-term gastrointestinal morbidity of the offspring according to maternal celiac (log rank, *p* < 0.001).

After excluding cases of perinatal mortality, the study population included 242,323 offspring, among them 210 offspring of mothers with celiac disease. Offspring born to mothers with celiac disease had higher rates of long-term gastrointestinal morbidity (9.5% vs. 5.4% *p* = 0.008) compared to offspring of mother without celiac disease. Higher rates of celiac disease (2.4% vs. 0.4%, *p* < 0.001) and gastro-duodenal disease (2.4% vs. 0.5%, *p* < 0.001) wereseen among offspring ofwomen with celiac disease, compared to offspring of women without celiac disease (Table 3). Likewise, the Kaplan–Meier survival curve demonstrated higher cumulative incidence of gastrointestinal morbidity among offspring of mothers with celiac disease (log rank test *p* < 0.001, Figure 1). Five separate Cox multivariable regression models were constructed, controlling each one for different confounders such as maternal age, birth weight, hypertensive disorders, cesarean section, and preterm delivery. All models have demonstrated that maternal celiac disease was independently associated with long-term gastrointestinal morbidity of the offspring (Table 4).

## 4. Discussion

Celiac disease is a relatively common disease that shows an increased prevalence in females, particularly during fertile period [7]. Our study has shown higher rates of adverse perinatal outcomes among women with celiac disease, such as several cesarean delivery and low birthweight. Furthermore, offspring born to mothers with celiac disease had higher rates of long-term total gastrointestinal morbidity and specifically higher rates of celiac disease and gastro-duodenal disease compared to offspring of women without celiac disease.

The association between women with celiac disease and lowbirth weight has been previously documented [11,16]. Ludvigsson et al. reported that undiagnosed celiac disease, before conception, was associated with an increased risk of low birthweight (<2500 g), very low birthweight (<1500 g), and preterm birth [11]. In their retrospective cohort study, Norgard et al. found an increased risk of low birthweight in offspring of women with celiac disease whose birth occurred before hospitalization of the mother [16]. Low birth-weight may be a result of nutrient deficiencies, seen in pregnant women with celiac disease, including iron, folat, and vitamin B12 deficiencies [16,17]. Anti-tissuetranglutaminase (t-TG) antibodies are among the most sensitive serologic test in the screening of celiac disease. The levels of anti-t-TG antibodies reflect the degree of mucosal damage of small bowel and may be correlated with the degree of fetal growth restriction [17]. Other studies suggest that anti-t-TG antibodies may not only serve as a diagnostic marker of celiac disease but may also be involved in impair placental development and function. It may be related to pregnancy complications such as fetal growth restriction and low birth weight [17,18,19]. Nevertheless, as we did not have information regarding the compliance to gluten-free diet of the patients, we can assume that some mothers did not strictly follow the recommendation for gluten-free diet.

Our study also demonstrated significantly higher rates of preterm deliveries. Nevertheless, using a GEE model controlling for maternal age and parity, the association lost its significance. Other studies have assessed the association between maternal celiac disease and preterm delivery. In a retrospective cohort study, 329 women with celiac disease and 641 women without the disease completed an anonymous online survey, answering 43 questions regarding their menstrual history, fertility, and pregnancy outcomes. Women with celiac disease had higher rates of preterm delivery (23.6% vs. 15.9% *p* = 0.02) [20]. A large Danish study investigated pregnancy outcome of women with appropriately treated celiac who were compared to women with undiagnosed celiac disease (that was considered as untreated). Women with undiagnosed celiac disease had higher rates of preterm delivery (OR 1.33; 95% CI 1.02–1.72) and small for gestation age infants (OR 1.54; 95% CI 1.17–2.03) compared to women with celiac that was appropriately treated [21].

In agreement to our study, Tata et al. demonstrated an increased prevalence of cesarean section in women with celiac disease (OR 1.33; 95% CI 1.03–1.70). In their study, increased rates of cesarean delivery were mostly seen among older parturient and were more likely be related to socioeconomic advantage of women with celiac disease [8]. Moleski et al. found that women with celiac disease were more likely to deliver in cesarean delivery (a rate as high as 31.2%), but the difference was not statistically significant (*p* = 0.24) [20].

Celiac disease was found to be associated with changes in gut microbiome in patients with this inflammatory disorder [22]. Moreover, a genetic predisposition to celiac has a strong influence on the intestinal colonization of infants in families at risk of developing celiac disease. Olivares et al. [23] demonstrated that carrying the HLA-DQ2 haplotypes influences the early gut microbiota composition. The imbalance in intestinal microbiome that is found during infancy among those with high genetic risk of developing celiac disease might have an effect on other gastrointestinal diseases in offspring of mothers with celiac disease.

Our study’s major strength is the fact that our hospital is the only hospital serving the entire population of Southern Israel (the Negev). The hospital provides medical health care for mothers and their offspring; thus, as long as the patient and her child live in the area, they would most probably be diagnosed and treated in this hospital. Even though our findings regarding higher rates of adverse perinatal outcomes correlate with previous studies concerning women with celiac disease, the study is the first to investigate the long-term gastrointestinal morbidity in offspring of women with celiac disease.

Our study also has limitations. First, our main limitation is the lack of available clinical data from the patients. We were not able to differentiate between treated mothers with celiac to those who are not treated. Details about time of diagnosis, duration of the disease, and its severity are not known. Studies showed that undiagnosed celiac disease during pregnancy increased the risk of intrauterine growth retardation and low birth weight relative to controls, whereas this risk disappeared if celiac disease was diagnosed and treated before pregnancy. As mentioned above, we demonstrated an association between women with celiac disease and low birth weight; however, information regarding the years on a gluten-free diet at pregnancy and the age of diagnosis of the maternal celiac disease are missing. Furthermore, we followed these children only up to 18 years of age. It has been suggested that there is a correlation between the duration of gluten exposure and development of an immune response to gluten in genetically susceptible individuals [14]. Therefore, the rate of gastrointestinal morbidity in offspring of mothers with celiac might be higher than we showed.

In conclusion, our study was the first to investigate the long-term gastrointestinal morbidity in offspring of mothers with celiac disease. We showed a significant increase in gastro-duodenal disease, celiac disease, and total gastrointestinal morbidity. In addition, we demonstrated higher rates of adverse perinatal outcomes such as cesarean delivery and low birth weight in mothers with celiac disease. Screening tests are not routinely indicated and are conflicting in the literature [7]. Additional studies should be done before recommending screening for celiac disease during pregnancy and should investigate the effect of treatment on the short and long-term perinatal outcome.

## Figures and Tables

**Table 1:** Characteristics in study population.

	Mothers with Celiac (*n* = 212)	Mothers without Celiac (*n* = 243,470)	*p*-Value
Maternal age, Years (mean ± SD)	28.8 ± 5.5	28.1 ± 5.8	0.081
Parity (%)			
1	24.1	23.6	0.470
2–4	54.2	51.1	
≥5	21.7	25.3	
Ethnicity			
Jewish (%)	42.5	47.3	0.154
Bedouin (%)	57.5	52.7	

**Table 2:** The association between maternal celiac disease and adverse perinatal outcome; univariable analysis and results from generalized estimation equation models controlling for maternal age and parity.

Characteristic	Mother with CeliacDisease (*n* = 212)	Mother without CeliacDisease (*n* = 243,470)	OR (95%CI)	*p*-Value	aOR (95% CI)	*p*-Value
Hypertensive disorders * (%)	0.5	5.0	0.08 (0.01–0.63)	0.002	0.1 (0.03–0.50)	0.003
Preterm delivery (%)	10.4	6.9	1.5 (1.01–2.44)	0.043	1.5 (0.92–2.48)	0.103
Cesarean section (%)	18.4	13.5	1.4 (1.01–2.03)	0.039	1.4 (1.02–2.07)	0.035
Low birth weight (%)	14.2	6.7	2.2 (1.55–3.36)	<0.001	2.2 (1.50–3.47)	<0.001
Low Apgar score at 5th minute (%)	1.4	2.3	0.6 (0.19–1.94)	0.407	0.5 (0.16–1.96)	0.372
Perinatal mortality ** (%)	0.9	0.5	1.7 (0.42–6.94)	0.438	1.5 (0.33–6.92)	0.584

CI, confidence interval; OR, odds ratio. * Hypertensive disorders in pregnancy includegestational hypertension, preeclampsia, eclampsia, and chronic hypertension. ** Perinatal mortality includes intrauterine fetal death, intrapartum death, and postpartum death.

**Table 3:** Association between maternal celiac disease and long-term gastrointestinal morbidity of the offspring.

Offspring Long-Term Gastrointestinal Morbidity N	Maternal Celiac Disease (*n* = 210) N (%)	No CeliacDisease (*n* = 242,132) N (%)	*p*-Value
Esophageal disease, 524	0 (<0.01)	524 (0.2)	0.500
Gastro-duodenal disease 1292	5 (2.4)	1287 (0.5)	<0.001
Appendix disease 1468	1(0.5)	1467(0.6)	0.809
Ano-rectal disease 476	0 (<0.01)	476(0.2)	0.520
Umbilical or inguinal hernia 3747	6 (2.9)	3468 (1.4)	0.083
IBD 4126	3(1.4)	4123 (1.7)	0.759
Functional colon disease 377	0 (0.0%)	377 (0.2)	0.567
Hemorrhoids 260	1(0.5)	259 (0.1)	0.102
Celiac disease 940	5(2.4)	935(0.4)	<0.001
Total gastrointestinal hospitalizations 13,023	20 (9.5)	13003(5.4)	0.008

**Table 4:** Proportional hazards model, to predict offspring long-term gastrointestinal morbidity.

		Adjusted HR	95% CI of HR	*p*-Value
Model 1	Maternal celiac disease (vs. no celiac)	2.5	1.6–3.9	<0.001
	Maternal age (years)	0.99	0.98–0.99	<0.001
Model 2	Maternal celiac disease (vs. no celiac)	2.5	1.6–3.8	<0.001
	Birth weight (grams)	1.0	1.00–1.00	<0.001
Model 3	Maternal celiac disease (vs. no celiac)	2.5	1.6–3.9	<0.001
	Hypertensive disorders	1.07	0.99–1.16	0.05
Model 4	Maternal celiac disease (vs. no celiac)	2.5	1.6–3.9	<0.001
	Cesarean section	1.29	1.23–1.35	<0.001
Model 5	Maternal celiac disease (vs. no celiac)	2.5	1.6–3.9	<0.001
	Preterm delivery (<37 weeks)	1.459	1.37–1.54	<0.001

## Supplementary Materials

The following are available online at https://www.mdpi.com/2077-0383/8/11/1924/s1, Table S1: International classification of diseases, ICD-9 codes for gastrointestinal morbidities accounted for.
