# Supplementary material for: Perinatal Outcome and Long-Term Gastrointestinal Morbidity of Offspring of Women with Celiac Disease
Source: J Clin Med. 2019 Nov 8;8(11):1924. doi: 10.3390/jcm8111924 (PMC6912641; doi:10.3390/jcm8111924)
Supplement: Supplementary file 1 [file jcm-08-01924-s001.pdf]

***Supplemental Table:*** *International classification of diseases, ICD-9 codes for gastrointestinal morbidities accounted for.*

| ***ICD-9 code*** | **Group** | **Diagnosis description** |
|---|---|---|
| *5300* | ***Esophageal*** | ACHALASIA AND CARDIOSPASM |
| *5301* | | ESOPHAGITIS |
| *5302* | | ULCER OF ESOPHAGUS |
| *5303* | | STRICTURE AND STENOSIS OF ESOPHAGUS |
| *5304* | | PERFORATION OF ESOPHAGUS |
| *5305* | | DYSKINESIA OF ESOPHAGUS |
| *5307* | | GASTROESOPHAGEAL LACERATION-HEMORRHAGE SYNDROME |
| *5308* | | OTHER SPECIFIED DISORDERS OF ESOPHAGUS |
| *5309* | | UNSPECIFIED DISORDER OF ESOPHAGUS |
| *53010* | | ESOPHAGITIS, UNSPECIFIED |
| *53011* | | REFLUX ESOPHAGITIS |
| *53012* | | ACUTE ESOPHAGITIS |
| *53013* | | EOSINOPHILIC ESOPHAGITIS |
| *53020* | | ULCER OF ESOPHAGUS WITHOUT BLEEDING |
| *53021* | | ULCER OF ESOPHAGUS WITH BLEEDING |
| *53081* | | ESOPHAGEAL REFLUX |
| *53084* | | TRACHEOESOPHAGEAL FISTULA |
| *53085* | | BARRETT'S ESOPHAGUS |
| *53089* | | OTHER SPECIFIED DISORDERS OF ESOPHAGUS |
| *5310* | ***Gastroduodenal*** | ACUTE GASTRIC ULCER WITH HEMORRHAGE |
| *5316* | | CHR.OR UNSP.GASTRIC ULCER WITH HEMORRHAGE AND PERFORATION |
| *5326* | | CHR./UNSP.DUODENAL ULCER WITH HEMORRHAGE AND PERFORATION |
| *5334* | | CHR.OR UNSPECIFIED PEPTIC ULCER WITH HEMORRHAGE |
| *5354* | | OTHER SPECIFIED GASTRITIS |
| *5355* | | UNSPECIFIED GASTRITIS AND GASTRODUODENITIS |
| *5363* | | GASTROPARESIS |

| | | |
|---|---|---|
| *5370* | | ACQUIRED HYPERTROPHIC PYLORIC STENOSIS |
| *5373* | | OTHER OBSTRUCTION OF DUODENUM |
| *5374* | | FISTULA OF STOMACH OR DUODENUM |
| *5379* | | UNSPECIFIED DISORDER OF STOMACH AND DUODENUM |
| *53110* | | AC.GASTRIC ULCER WITH PERFORATION WITHOUT OBSTRUCTION |
| *53130* | | AC.GASTRIC ULCER WITHOUT HEMORR.PERFOR.OR OBSTRUCTION |
| *53140* | | CHR.OR UNSP.GASTRIC ULCER WITH HEMOR.WITHOUT OBSTRUCTION |
| *53150* | | CHR.OR UNSP.GASTRIC ULCER WITH PERF.WITHOUT OBSTRUCTION |
| *53170* | | CHR.GASTRIC ULCER WITHOUT HEMOR./PERF.WITHOUT OBSTRUCTION |
| *53190* | | GASTRIC ULCER,UNSP.WITHOUT HEMOR.PERFOR.WITHOUT OBSTRUCTION |
| *53201* | | ACUTE DUODENAL ULCER WITH HEMORRHAGE, WITH OBSTRUCTION |
| *53210* | | AC.DUODENAL ULCER WITH PERF.WITHOUT MENTION OF OBSTRUCTION |
| *53250* | | CHR./UNSP.DUODENAL ULCER WITH PERF.WITHOUT OBSTRUCTION |
| *53270* | | CHR.DUODENAL ULCER WITHOUT HEMORR./PERF.WITHOUT OBSTRUCTION |
| *53290* | | DUODENAL ULCER,AC./CHR.WITHOUT HEMORR.PERFOR.OR OBSTRUCTION |
| *53350* | | CHR./UNSP.PEPTIC ULCER WITH PERF.WITHOUT OBSTRUCTION |
| *53500* | | AC. GASTEITIS WITHOUT MENTION OF HEMORRHAGE |
| *53501* | | ACUTE GASTRITIS WITH HEMORRHAGE |
| *53510* | | ATROPHIC GASTRITIS WITHOUT MENTION OF HEMORRHAGE |
| *53511* | | ATROPHIC GASTRITIS WITH HEMORRHAGE |
| *53520* | | GASTRIC MUCOSAL HYPERTROPHY WITHOUT MENTION OF |

| | | |
|---|---|---|
| | | HEMORRHAGE |
| *53530* | | ALCOHOLIC GASTRITIS WITHOUT MENTION OF HEMORRHAGE |
| *53540* | | OTHER SPECIFIED GASTRITIS WITHOUT MENTION OF HEMORRHAGE |
| *53550* | | UNSP. GASTRITIS/GASTRODUODENITIS WITHOUT HEMORRHAGE |
| *53551* | | UNSP. GASTRITIS/GASTRODUODENITIS WITH HEMORRHAGE |
| *53560* | | DUODENITIS WITHOUT MENTION OF HEMORRHAGE |
| *53789* | | OTHER SPECIFIED DISORDERS OF STOMACH AND DUODENUM |
| *5368* | ***Functional*** | DYSPEPSIA AND OTHER SPECIFIED DISORDERS OF FUNCTION OF STOMACH |
| *5369* | | UNSPECIFIED FUNCTIONAL DISORDER OF STOMACH |
| *541* | ***Appendix*** | APPENDICITIS, UNQUALIFIED |
| *5400* | | ACUTE APPENDICITIS WITH GENERALIZED PERITONITIS |
| *5401* | | ACUTE APPENDICITIS WITH PERITONEAL ABSCESS |
| *5409* | | ACUTE APPENDICITIS WITHOUT MENTION OF PERITONITIS |
| *5430* | | HYPERPLASIA OF APPENDIX (LYMPHOID) |
| *5439* | | OTHER AND UNSPECIFIED DISEASES OF APPENDIX |
| *550* | ***Hernia-inguinal umbilical abdominal wall*** | INGUINAL HERNIA |
| *5509* | | INGUINAL HERNIA, WITHOUT MENTION OF OBSTRUCTION OR GANGRENE |
| *5510* | | FEMORAL HERNIA WITH GANGRENE |
| *5521* | | UMBILICAL HERNIA WITH OBSTRUCTION |
| *5523* | | DIAPHRAGMATIC HERNIA WITH OBSTRUCTION |
| *5529* | | HERNIA OF UNSPECIFIED SITE, WITH OBSTRUCTION |
| *5531* | | UMBILICAL HERNIA |
| *5531* | | UMBILICAL HERNIA WITHOUT MENTION OF OBSTRUCTION OR GANGRENE |
| *5533* | | DIAPHRAGMATIC HERNIA WITHOUT MENTION OF OBSTRUCTION OR GANGRENE |
| *5538* | | HERNIA OF OTHER SPECIF.SITES WITHOUT OBSTRUCTION OR GANGRENE |

| | | |
|---|---|---|
| *5539* | | HERNIA OF UNSP.SITE WITHOUT OBSTRUCTION OR GANGRENE |
| *55000* | | UNILATERAL OR UNSPECIFIED INGUINAL HERNIA, WITH GANGRENE |
| *55002* | | BILATERAL INGUINAL HERNIA, WITH GANGRENE |
| *55010* | | UNILAT.OR UNSP.INGUINAL HERNIA, WITH OBST.WITHOUT GANGRENE |
| *55011* | | REC.UNIL.OR UNSP.INGUINAL HERNIA WITH OBST.WITHOUT GANGRENE |
| *55090* | | UNIL./UNSP.INGUINAL HERNIA, WITHOUT OBSTR.OR GANGRENE |
| *55091* | | REC.UNIL.OR UNSP.INGUINAL HERNIA WITHOUT OBSTR.OR GANGRENE |
| *55092* | | BILATERAL INGUINAL HERNIA, WITHOUT OBSTRUCTION OR GANGRENE |
| *55200* | | UNILATERAL OR UNSPECIFIED FEMORAL HERNIA WITH OBSTRUCTION |
| *55229* | | OTHER VENTRAL HERNIA WITH OBSTRUCTION |
| *55300* | | UNILAT./UNSPEC.FEMORAL HERNIA WITHOUT OBSTRUCTION OR GANGRENE |
| *55302* | | BILAT.FEMORAL HERNIA WITHOUT OBSTRUCTION OR GANGRENE |
| *55320* | | UNSP.VENTRAL HERNIA WITHOUT OBSTRUCTION OR GANGRENE |
| *55321* | | INCISIONAL HERNIA WITHOUT MENTION OF OBSTRUCTION OR GANGRENE |
| *55329* | | VENTRAL HERNIA WITHOUT MENTION OF OBSTRUCTION OR GANGRENE |
| *558* | ***IBD*** | OTHER NONINFECTIOUS GASTROENTERITIS AND COLITIS |
| *5550* | | REGIONAL ENTERITIS OF SMALL INTESTINE |
| *5551* | | REGIONAL ENTERITIS OF LARGE INTESTINE |
| *5559* | | REGIONAL ENTERITIS OF UNSPECIFIED SITE |
| *5562* | | ULCERATIVE (CHRONIC) PROCTITIS |

| | | |
|---|---|---|
| *5566* | | UNIVERSAL ULCERATIVE (CHRONIC) COLITIS |
| *5569* | | ULCERATIVE COLITIS, UNSPECIFIED |
| *5581* | | GASTROENTERITIS AND COLITIS DUE TO RADIATION |
| *5583* | | ALLERGIC GASTROENTERITIS AND COLITIS |
| *5589* | | OTHER & UNSPEC.NONINFECTIOUS GASTROENTERITIS & COLITIS |
| *5589* | | OTHER AND UNSPEC.NONINFECTIOUS GASTROENTERITIS AND COLITIS |
| *5570* | ***Vascular*** | ACUTE VASCULAR INSUFFICIENCY OF INTESTINE |
| *5571* | | CHRONIC VASCULAR INSUFFICIENCY OF INTESTINE |
| *5579* | | UNSPECIFIED VASCULAR INSUFFICIENCY OF INTESTINE |
| *V1272* | | PERSONAL HISTORY OF COLONIC POLYPS |
| *5640* | ***Colonic-functional*** | CONSTIPATION |
| *5641* | | IRRITABLE BOWEL SYNDROME |
| *5646* | | ANAL SPASM |
| *5647* | | MEGACOLON, OTHER THAN HIRSCHSPRUNG'S |
| *5649* | | UNSPECIFIED FUNCTIONAL DISORDER OF INTESTINE |
| *56400* | | CONSTIPATION, UNSPECIFIED |
| *56481* | | NEUROGENIC BOWEL |
| *56489* | | OTHER FUNCTIONAL DISORDERS OF INTESTINE |
| *5671* | ***Peritoneal*** | PNEUMOCOCCAL PERITONITIS |
| *5672* | | OTHER SUPPURATIVE PERITONITIS |
| *5679* | | UNSPECIFIED PERITONITIS |
| *5689* | | UNSPECIFIED DISORDER OF PERITONEUM |
| *56721* | | PERITONITIS (ACUTE) GENERALIZED |
| *56722* | | PERITONEAL ABSCESS |
| *56723* | | SPONTANEOUS BACTERIAL PERITONITIS |
| *56729* | | OTHER SUPPURATIVE PERITONITIS |
| *56731* | | PSOAS MUSCLE ABSCESS |
| *56882* | | PERITONEAL EFFUSION (CHRONIC) |
| *56889* | | OTHER SPECIFIED DISORDERS OF PERITONEUM |
| *566* | ***Anorectal*** | ABSCESS OF ANAL AND RECTAL REGIONS |
| *5650* | | ANAL FISSURE |

| | | |
|---|---|---|
| *5651* | | ANAL FISTULA |
| *5690* | | ANAL AND RECTAL POLYP |
| *5691* | | RECTAL PROLAPSE |
| *5692* | | STENOSIS OF RECTUM AND ANUS |
| *5693* | | HEMORRHAGE OF RECTUM AND ANUS |
| *56941* | | ULCER OF ANUS AND RECTUM |
| *56942* | | ANAL OR RECTAL PAIN |
| *56949* | | OTHER SPECIFIED DISORDERS OF RECTUM AND ANUS |
| *5720* | ***Hepatitis*** | ABSCESS OF LIVER |
| *5713* | | ALCOHOLIC LIVER DAMAGE, UNSPECIFIED |
| *5716* | | BILIARY CIRRHOSIS |
| *57142* | | AUTOIMMUNE HEPATITIS |
| *570* | | ACUTE AND SUBACUTE NECROSIS OF LIVER |
| *573* | | OTHER DISORDERS OF LIVER |
| *2774* | | DISORDERS OF BILIRUBIN EXCRETION |
| *5715* | | CIRRHOSIS OF LIVER WITHOUT MENTION OF ALCOHOL |
| *5718* | | OTHER CHRONIC NONALCOHOLIC LIVER DISEASE |
| *5722* | | HEPATIC COMA |
| *5722* | | HEPATIC ENCEPHALOPATHY |
| *5723* | | PORTAL HYPERTENSION |
| *5724* | | HEPATORENAL SYNDROME |
| *5728* | | OTHER SEQUELAE OF CHRONIC LIVER DISEASE |
| *5730* | | CHRONIC PASSIVE CONGESTION OF LIVER |
| *5731* | | HEPATITIS IN VIRAL DISEASES CLASSIFIED ELSEWHERE |
| *5733* | | HEPATITIS, UNSPECIFIED |
| *5738* | | OTHER SPECIFIED DISORDERS OF LIVER |
| *5739* | | UNSPECIFIED DISORDER OF LIVER |
| *57140* | | CHRONIC HEPATITIS, UNSPECIFIED |
| *57149* | | OTHER CHRONIC HEPATITIS |
| *5600* | ***Surgical-*** | INTUSSUSCEPTION |
| *5601* | ***obstruction,*** | PARALYTIC ILEUS |
| *5602* | ***intussusception,*** | VOLVULUS |
| *5608* | ***volvulus,*** | OTHER SPECIFIED INTESTINAL OBSTRUCTION |

| | | |
|---|---|---|
| *5609* | ***perforation*** | UNSPECIFIED INTESTINAL OBSTRUCTION |
| *5642* | | POSTGASTRIC SURGERY SYNDROMES |
| *5680* | | PERITONEAL ADHESIONS (POSTOPERATIVE)(POSTINFECTION) |
| *5792* | | BLIND LOOP SYNDROME |
| *5793* | | OTHER AND UNSPECIFIED POSTSURGICAL NONABSORPTION |
| *56030* | | IMPACTION OF INTESTINE, UNSPECIFIED |
| *56039* | | OTHER IMPACTION OF INTESTINE |
| *56081* | | INTESTINAL OR PERITONEAL ADHESIONS WITH OBSTRUCTION |
| *56081* | | INTESTINAL/PERITONEAL ADHESIONS+OBSTRUCTION (POST-OPERATIVE/INFEC |
| *56089* | | OTHER SPECIFIED INTESTINAL OBSTRUCTION |
| *56210* | | DIVERTICULOSIS OF COLON (WITHOUT HEMORRHAGE) |
| *56211* | | DIVERTICULITIS OF COLON (WITHOUT HEMORRHAGE) |
| *56881* | | HEMOPERITONEUM (NONTRAUMATIC) |
| *56960* | | COLOSTOMY & ENTEROSTOMY COMPLICATION, UNSP. |
| *56961* | | INFECTION OF COLOSTOMY OR ENTEROSTOMY |
| *56962* | | MECHANICAL COMPLICATION OF COLOSTOMY & ENTEROSTOMY |
| *56969* | | OTHER COMPLICATION OF COLOSTOMY & ENTEROSTOMY |
| *56981* | | FISTULA OF INTESTINE, EXCLUDING RECTUM AND ANUS |
| *56983* | | PERFORATION OF INTESTINE |
| *5741* | ***Cholecystitis - lithalsas*** | CHOLELITHIASIS WITH OTHER CHOLECYSTITIS |
| *5750* | | ACUTE CHOLECYSTITIS |
| *5758* | | OTHER SPECIFIED DISORDERS OF GALLBLADDER |
| *5761* | | CHOLANGITIS |
| *5762* | | OBSTRUCTION OF BILE DUCT |
| *5768* | | OTHER SPECIFIED DISORDERS OF BILIARY TRACT |
| *57400* | | CHOLELITHIASIS + AC.CHOLECYSTITIS, WITHOUT OBSTRUCTION |
| *57410* | | CHOLELITHIASIS + OTHER CHOLECYSTITIS, WITHOUT OBSTRUCTION |
| *57411* | | CHOLELITHIASIS + OTHER CHOLECYSTITIS + OBSTRUCTION |

| | | |
|---|---|---|
| *57420* | | CHOLELITHIASIS WITHOUT CHOLECYSTITIS OR OBSTRUCTION |
| *57440* | | CALCULUS OF BILE DUCT + OTHER CHOLECYSTITIS,WITHOUT OBSTRUCTION |
| *57450* | | CALCULUS OF BILE DUCT WITHOUT CHOLECYSTITIS OR OBSTRUCTION |
| *57451* | | CALCULUS OF BILE DUCT WITHOUT CHOLECYSTITIS,WITH OBSTRUCTION |
| *57470* | | CALCULUS:GALLBLADDER & BILE DUCT+OTHER CHOLECYSTITIS,WITHOUT OBST |
| *57510* | | CHOLECYSTITIS, UNSP. |
| *57511* | | CHOLECYSTITIS, CHRONIC |
| *5770* | ***Pancreatic*** | ACUTE PANCREATITIS |
| *5771* | | CHRONIC PANCREATITIS |
| *5772* | | CYST AND PSEUDOCYST OF PANCREAS |
| *5778* | | OTHER SPECIFIED DISEASES OF PANCREAS |
| *5794* | | PANCREATIC STEATORRHEA |
| *5790* | ***Celiac*** | CELIAC DISEASE |
| *5791* | | TROPICAL SPRUE |
| *5798* | | OTHER SPECIFIED INTESTINAL MALABSORPTION |
| *5799* | | UNSPECIFIED INTESTINAL MALABSORPTION |
| *5780* | ***Hemorrhoids*** | HEMATEMESIS |
| *5781* | | BLOOD IN STOOL (MELENA) |
| *5781* | | MELENA |
| *5789* | | HEMORRHAGE OF GASTROINTESTINAL TRACT, UNSPECIFIED |
| *5699* | ***Other*** | UNSPECIFIED DISORDER OF INTESTINE |
| *9974* | | DIGESTIVE SYSTEM COMPLICATIONS N.E.C. |
| *9974* | | GASTROINTESTINAL COMPLICATIONS, NOT ELSEWHERE CLASSIFIED |
| *56989* | | OTHER SPECIFIED DISORDERS OF INTESTINES |
| *60622* | | MILIA |
